# Overexpression of suppressor of zest 12 is associated with cervical node metastasis and unfavorable prognosis in tongue squamous cell carcinoma

**DOI:** 10.1186/s12935-017-0395-9

**Published:** 2017-02-13

**Authors:** Huijun Hu, Yi Wang, Zhongwu Li, Yumin Zhu, Wei Zhang, Dongmiao Wang, Tangyi Lin, Jianrong Yang, Yanling Wang, Jie Cheng

**Affiliations:** 1 Jiangsu Key Laboratory of Oral Diseases, Nanjing Medical University, Nanjing, 210029 People’s Republic of China; 2 Department of Oral and Maxillofacial Surgery, Affiliated Stomatological Hospital, Nanjing Medical University, 136, Hanzhong Road, Nanjing, 210029 People’s Republic of China; 3 Department of Oral Pathology, Affiliated Stomatological Hospital, Nanjing Medical University, Nanjing, 210029 People’s Republic of China

**Keywords:** SUZ12, Polycomb repressive complex, Tongue squamous cell carcinoma, Prognosis

## Abstract

**Objective:**

Increased expression of suppressor of zest 12 (SUZ12), a core component of the polycomb repressive complex 2, contributes to human tumorigenesis and associates with patient prognosis. In the present study, we sought to investigate the expression of SUZ12 and its clinicopathological significance in primary tongue squamous cell carcinoma (TSCC).

**Methods:**

The expression of SUZ12 protein was determined by immunohistochemistry in clinical samples from a retrospective cohort of 72 patients with primary TSCC who were treated at our institution from Jan. 2007 to Dec. 2013. The potential associations between SUZ12 abundance and multiple clinicopathological parameters were assessed by Chi square test. Moreover, the effect of SUZ12 expression on patients’ survival was further estimated by Kaplan–Meier and Cox regression analyses.

**Results:**

Our immunohistochemical staining data revealed aberrant overexpression of SUZ12 in a large subset of TSCC as compared to normal tongue mucosa. Elevated SUZ12 was found to be significantly associated with cervical nodes metastasis (*P* = 0.0325) and reduced overall as well as disease-free survival (Log-rank test, *P* = 0.0225, 0.0179, respectively). Both univariate and multivariate Cox regression analysis identified the expression status of SUZ12 (low/high) as an important independent prognostic factor for patients’ survival.

**Conclusions:**

Our data reveal that aberrant SUZ12 overexpression is associated with cervical nodes metastasis and reduced survival in TSCC. These findings suggest that SUZ12 might play critical roles during tongue tumorigenesis and serve as a novel biomarker with diagnostic and prognostic significance.

## Background

Oral cancer is one of the most common solid malignancy worldwide, accounting for approximately 3% of all malignancies in both sexes. Several etiologic risks including human papillomavirus infection, smoking, and heavy alcohol consumption have been increasingly recognized for oral carcinogenesis [1, 2]. The overweighing majority of oral cancer is classified as squamous cell carcinoma (SCC) and mostly arises from tongue followed by mouth floor, buccal as well as gingiva [1, 2]. Thus, tongue SCC (TSCC) is a representative cancer subtype of OSCC and accounts for a large fraction of OSCC cases. The past decades have witnessed tremendous progress in multi-modal therapy against TSCC. However, the long-term survival for patients with TSCC is not markedly improved, especially for those with advanced diseases [3]. These facts underscore the highly aggressive nature of TSCC and the unresolved challenge for diagnostic and therapeutic management of this malignancy in the clinic. Until now, few biomarkers have been equivocally established for diagnostic and prognostic management of tongue cancer. Therefore, the identification of the new biomarkers and therapeutic targets for TSCC is paramount and urgent for clinicians to improve the patients’ prognosis.

Epigenetic abnormality is a hallmark of human cancer and greatly contributes to cancer initiation and progression [4]. Mounting evidence has established that several epigenetic modulators have been identified as key mediators driving tumorigenesis by serving as pro-oncogenes or tumor suppressor genes. Moreover, these epigenetic modulators hold great promise as putative therapeutic targets against cancer largely owing to their pervasive roles in gene regulation as well as the inherent reversible nature of epigenetic alternations [5]. Among these epigenetic modulators underlying tumorigenesis, several members of polycomb group (PcG) proteins have stood out as essential participators of malignant transformation as well as promising targets for cancer therapeutic intervention. Previous studies have revealed that the PcG components usually assemble in stable multi-protein complexes together with additional factors to maintain their target genes in a transcriptionally repressive state [6]. In brief, two polycomb repressive complexes (PRC1 and PRC2) harbor multiple core members to execute their functions by histone modifications and in turn induce gene silencing [7]. Our previous studies and others have offered strong evidence that multiple members of PcG such as Bmi1 and EZH2 are bona fide oncogenes contributing to tumorigenesis in diverse sites throughout the whole body including tongue and their overexpression associates with cancer aggressiveness and poor prognosis in a broad spectrum of human cancer including tongue cancer [8–10]. Notably, suppressor of zest 12 (SUZ12) is one of the core component of PRC2, which is essential for PRC2-mediated gene silencing by generating trimethylation on lysine 27 residue of histone H3 (H3K27me3) [11]. However, the biological roles and associated molecular mechanisms of SUZ12 underlying tumor development are just beginning to be elucidated. For example, SUZ12 has been found to be frequently overexpressed in several solid cancers including colorectal, ovarian and non-small lung cancer, etc. [12–14]. Furthermore, its aberrant overexpression commonly associated with tumor aggressive behaviors, advanced clinicopathological features and decreased survival, thus suggesting potential roles of SUZ12 as a novel cancer biomarker and a putative oncogene [12–14]. However, to the best of our knowledge, the expression of SUZ12 and its clinicopathological significance in TSCC have not been established yet.

Herein, we sought to investigate the expression of SUZ12 protein in primary human TSCC specimens and identify potential relationship between its abundance and clinicopathological features as well as patients’ survival.

## Methods

### Patients and tissue specimens

A retrospective cohort of 72 patients with primary TSCC treated at our institution between Jan. 2007 and Dec. 2013 were enrolled. Written informed consent was obtained from these patients. Patient inclusion criteria were described as follows: (1) primary TSCC without any prior history of surgery, chemotherapy or radiotherapy; (2) patients underwent radical tumor resection and neck dissection (elective or therapeutic neck dissection as required); (3) detailed information available including epidemiologic, clinical, pathological and follow-up data. The archived tissue samples were retrieved and haematoxylin–eosin staining slides for each patient were further analyzed to confirm the previous diagnose based on the established histopathological criteria. Fifteen samples of healthy tongue mucosa were obtained from other non-cancer surgeries and histologically confirmed under microscope by senior oral pathologists. This study protocol was reviewed and approved by the Research Ethic Committee of Nanjing Medical University (2015-0126).

### Histopathological evaluation, clinicopathological categorization and immunohistochemical staining of SUZ12

The relevant clinicopathological parameters for each case including histological grade, TNM classification, clinical stage, etc. were determined similarly as we described before [8, 9, 15]. Immunohistochemical staining for SUZ12 was performed on 4-µm formalin-fixed, paraffin-embedded specimens using routine procedure. Briefly, tissue sections from representative paraffin blocks were deparaffinised in xylene and rehydrate through graded alcohols. Tissue slides were then processed in microwave heating in 10 mM citrate buffer (pH 6.0) for 15 min for antigen retrieval and 3% hydrogen peroxide for endogenous peroxidase inactivation. These sections were further incubated with primary antibody (anti-SUZ12, 1:200 dilution; Abcam, ab12073, USA) at 4 °C overnight and developed with 3.3′-diaminobenzidine and counterstained with haematoxylin. The immunoreactivity in each slide was assessed independently by two senior oral pathologists without knowledge about the relevant clinical and pathological data. Negative controls (without primary antibody incubation) were included in each staining run. Immunoreactivity was semi-quantitatively evaluated according to staining intensity and distribution using the immunoreactive score which was calculated as intensity score × proportion score as we reported previously [8, 15, 16]. Intensity score was defined as 0, negative; 1, weak; 2, moderate; 3, strong. The proportion score was defined as 0, negative; 1, <10%; 2, 11–50%; 3, 51–80%; 4, >80% positive cells. Therefore, the total score ranged from 0 to 12. Accordingly, the immunoreactivity of each slide was categorized into three subgroups based on the final score: 0, negative; 1–4, low expression; 4–12, high expression, similar as our previous reports [8, 15, 16].

### Statistical analyses

The associations between SUZ12 expression and various clinicopathological parameters of patients were evaluated using Chi square test. The survival rates of patients were estimated using Kaplan–Meier method and compared with Log-rank test. The prognostic analyses were performed by univariate and multivariate Cox regression models to determine the individual clinicopathological variables with patients’ overall or disease-free survival. *P* values <0.05 (two-sided) were considered statistically significant. All statistical analyses were performed using Graphpad Prism 5 (La Jolla, CA, USA) or SPSS 18.0 (Armonk, NY, USA).

## Results

### Clinicopathological characteristics and SUZ12 expression in primary TSCC

**Fig. 1:**
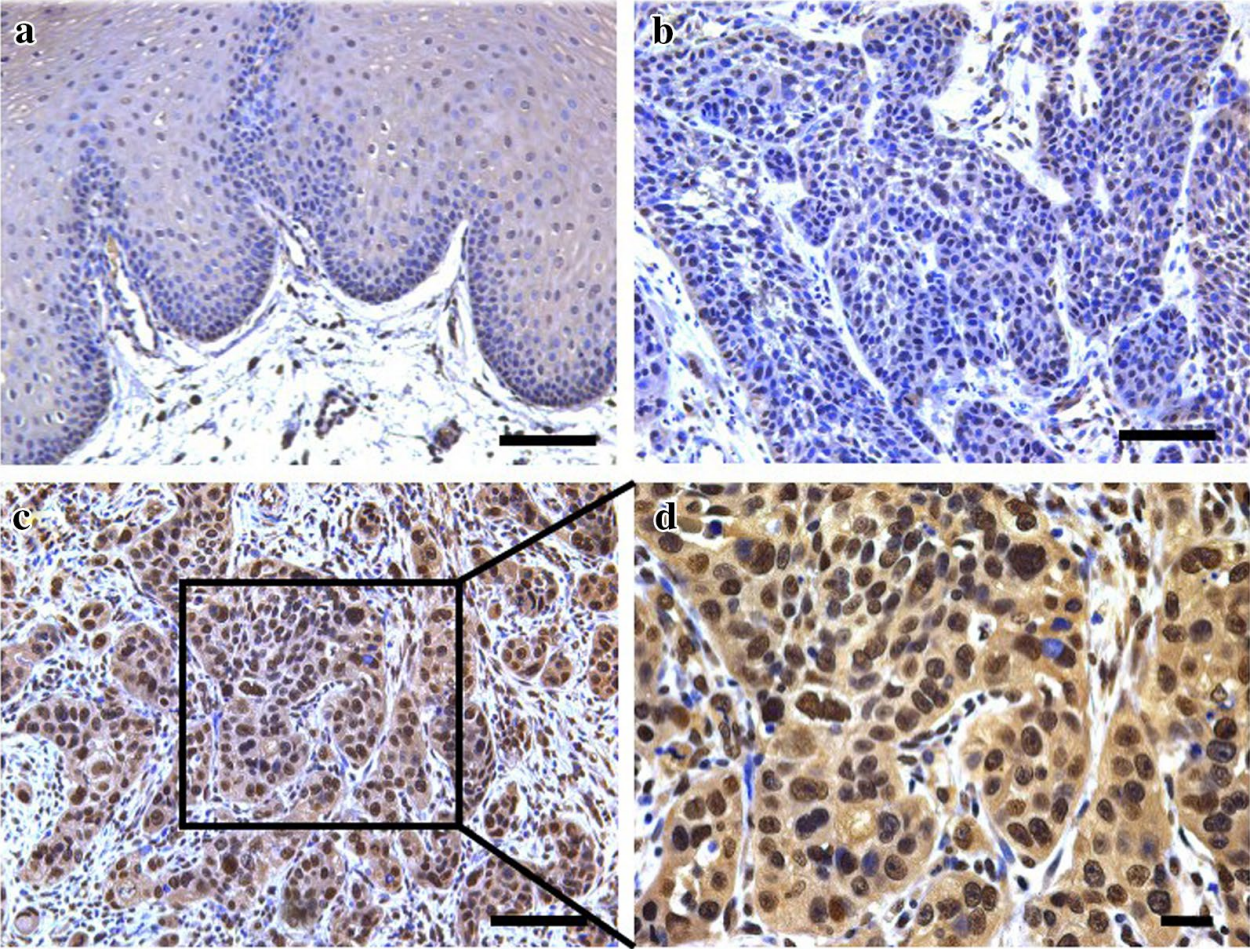
Immunohistochemical staining of SUZ12 in TSCC specimens and normal tongue mucosa. **a** Representative weak staining of SUZ12 (low expression) in normal human tongue mucosa (×200). Nuclei are counterstained with haematoxylin. **b** Representative weak staining of SUZ12 (low expression) in primary TSCC sample (×200). **c** Representative strong staining of SUZ12 (high expression) in primary TSCC sample (×200). **d** This image is magnified from the *black box area* in **c** (×400). SUZ12 expression is identified primarily in nuclei of cancer cells. *Scale bar* 100 μm

Previous studies have provided clues to support the notion that SUZ12 is usually overexpressed in several human cancers and might serve as a putative pro-oncogene associated with tumorigenesis [12, 13]. To examine SUZ12 expression in TSCC, we evaluated the expression levels of SUZ12 protein by immunohistochemical staining in a retrospective cohort of 72 primary TSCC samples. The epidemiologic information and relevant clinicopathological features (age, gender, smoking, alcohol use, pathological grade, tumor size, clinical stage, cervical node status) of these patients included were summarized in Table 1. In brief, 40 male and 32 female patients were enrolled with mean age 56.5 years (31–81 years). The follow-up period ranged from 28 to 95 months with average 52.8 months. Based on our immunohistochemistry scoring regime, SUZ12 protein abundance in these primary TSCC and normal tongue epithelial samples (n = 15) was categorized, respectively. As shown in Table 2, SUZ12 levels in TSCC samples were graded as negative (4), low (31) and high (37) expression, respectively. In parallel, its expression levels in their normal counterparts were divided into negative (5), low (7) and high (3), respectively. These data showed significant difference of SUZ12 expression pattern between TSCC and normal tongue mucosa, and also clearly indicated that SUZ12 was aberrantly overexpressed in a significant fraction of primary TSCC. The representative immunohistochemical staining of SUZ12 in TSCC and normal tongue mucosa was shown in Fig. 1. High SUZ12 expression was mainly identified in the nucleus and much less in the cytoplasm in cancer cells, whereas weak staining was observed in normal tongue epithelial cells. The detailed associations between SUZ12 expression and clinicopathological variables were further analyzed and shown in Table 1. There were no significant correlations between SUZ12 expression with patients’ gender, age, smoking, alcohol use, tumor size, pathological grade and clinical stage. However, significant association between SUZ12 abundance with cervical nodes metastasis was found with *P* value 0.0325 (Chi square test).

**Table 1:** The associations between SUZ12 expression and multiple clinicopathological parameters in primary TSCC

Clinicopathological parameters	Cases	SUZ12		*P* values
		Low^a^	High	
Gender		35	37	0.486
Male	40	21	19	
Female	32	14	18	
Age				
≤60	40	17	23	0.3429
>60	32	18	14	
Smoking				
No	37	19	18	0.6324
Yes	35	16	19	
Alcohol use				
No	40	22	18	0.2253
Yes	32	13	19	
Tumor size				
T1–T2	58	29	29	0.7683
T3–T4	14	6	8	
Pathological grade				
I	32	19	13	0.1542
II–III	40	16	24	
Cervical node metastasis				
N(0)	38	23	15	*0.0325*
N(+)	34	12	22	
Clinical stage				
I–II	36	21	15	0.1567
III–IV	36	14	22	

The number in italic indicate statistical significance with *p* values <0.05

^a^Both of patients with low and negative SUZ12 staining are stratified into low SUZ12 category for simplicity

**Table 2:** SUZ12 expression patterns in TSCC and normal tongue mucosa

	SUZ12 expression			*P* values
	Negative	Low	High	
Normal tongue mucosa	5	7	3	*0.0025*
TSCC	4	31	37	

The number in italic indicate statistical significance with *p* values <0.05

### SUZ12 expression associates with TSCC patients’ survival

**Fig. 2:**
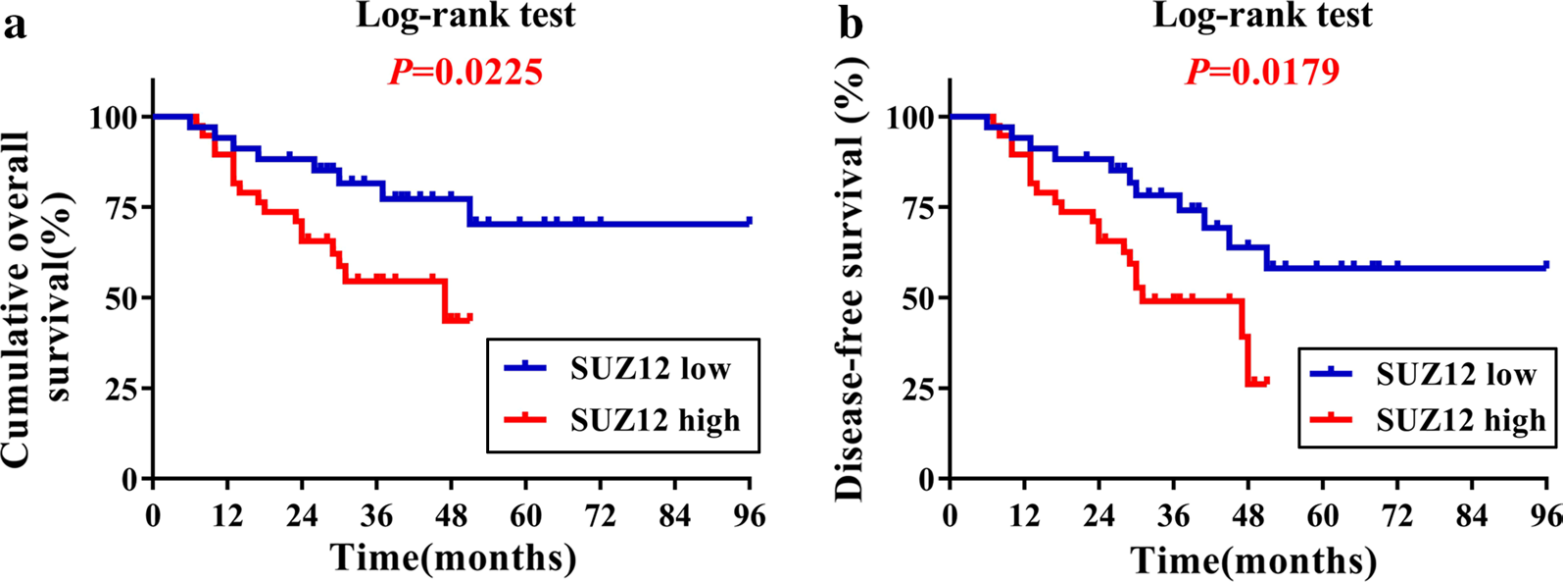
Kaplan–Meier graphs representing the probability of TSCC patients’ survival based on SUZ12 expression status. **a** High SUZ12 expression is significantly associated with reduced overall survival in TSCC patients. *P* value from Log-rank test is shown. **b** High SUZ12 expression is significantly associated with reduced disease-free survival in TSCC patients. *P* value from Log-rank test is shown

To reveal the potential prognostic value of SUZ12 expression in TSCC, we then attempted to evaluate the correlation between SUZ12 expression and patients’ survival. Until the last follow-up, 41 of 72 (56.9%) patients were alive and disease-free, 6 (8.3%) patients alive but with recurrence and/or cervical nodal metastases, 25 (34.7%) patients dying due to local recurrence, metastases, or other unrelated diseases. The results from Kaplan–Meier survival analyses indicated that high SUZ12 expression had an adverse prognostic impact on patients’ outcomes. In detail, as shown in Fig. 2, high SUZ12 abundance significantly associated with reduced overall survival and disease-free survival (Log-rank, *P* = 0.0225, 0.0179, respectively).

To further assess the clinical significance of SUZ12 expression as a prognostic predictor for patients with TSCC, the univariate and multivariate survival analyses (Cox proportional hazards regression model) were performed. As indicated in Table 3, the univariate survival analysis revealed that SUZ12 expression status and cervical node metastasis were significantly associated with overall survival (hazard ratio [HR] 3.023; 95% confidence interval [95% CI] 1.216–7.516; *P* = 0.017 for SUZ12; HR 1.759; 95% CI 1.240–3.291; *P* = 0.042 for cervical node metastasis, respectively), while other clinicopathological variable didn’t reach the statistical significance. To rule out the confounding factors, multivariate survival analysis was performed. In this Cox regression model, only the SUZ12 expression status was identified as an independent prognostic marker for the overall survival of patients with TSCC (HR 2.848; 95% CI 1.121–7.240; *P* = 0.028).

**Table 3:** Univariate and multivariate survival analyses for patients with primary TSCC using proportional hazards methods

Variable	Univariate survival analysis			Multivariate survival analysis		
	Hazard ratio	95% CI	*P* value	Hazard ratio	95% CI	*P* value
Gender (male, female)	1.651	0.739–3.688	0.221	1.428	0.599–3.405	0.422
Age (≤60, >60)	0.753	0.338–1.680	0.489	1.374	0.601–3.141	0.452
Smoking (no, yes)	1.253	0.653–1.835	0.524	1.372	0.665–1.732	0.536
Alcohol use (no, yes)	1.121	0.547–1.538	0.753	1.231	0.557–1.861	0.643
Tumor size (T1–T2, T3–T4)	1.331	0.528–3.357	0.544	1.447	0.505–4.147	0.491
Pathological grade (I, II–III)	1.258	0.490–1.994	0.117	1.246	0.444–3.496	0.676
Cervical nodal metastasis (N0, N+)	1.759	1.240–3.291	*0.042*	1.696	1.104–3.067	0.056
Clinical stage (I–II, III–IV)	1.203	0.688–1.824	0.095	0.922	0.524–1.622	0.778
SUZ12 expression (low^a^, high)	3.023	1.216–7.516	*0.017*	2.848	1.121–7.240	*0.028*

The numbers in italic indicate statistical significance with *p* values <0.05

^a^Both of patients with low and negative SUZ12 staining are stratified into low SUZ12 category for simplicity

## Discussion

The epigenetic modifiers, PRC1 and 2, regulate gene expression by modifying chromatin structure and are intricately implicated in various biological and pathological processes including stem cell plasticity, cell differentiation and proliferation as well as tumorigenesis [6, 17, 18]. Previous studies have suggested that the SUZ12, one of the core components of PRC2, might be an oncogene driving tumorigenesis and serve as a novel diagnostic biomarker and therapeutic target for cancer treatment [13, 18, 19]. Herein we investigated the expression pattern of SUZ12 in primary TSCC and determined its clinicopathological relevance and prognostic significance for patients with TSCC. Our findings reveal that SUZ12 is aberrantly overexpressed in a significant fraction of TSCC and its overexpression associates with aggressive clinicopathological features and unfavorable prognosis.

Tongue tumorigenesis in human is characterized by multiple and consecutive histopathological stages from normal epithelial to invasive SCC which is driven by oncogenes activation and tumor suppressor inactivation [2]. In particular, epigenetic inactivation of tumor suppressor genes contributes to initiation and progression of tongue cancer [20]. Among them, these chromatin modifiers like the polycomb members have been increasingly recognized as key players during tumorigenesis. They usually facilitate cancer initiation, overgrowth and metastasis, and also have been identified as therapeutic targets with remarkable translational potential [21]. We and others have provided evidence that several members of PcG such as EZH2 and Bmi1 are aberrantly upregulated in tongue cancer and associated with aggressiveness and poor prognosis [8, 10]. Notably, highly elevated SUZ12 has been found in bladder, gastric, non-small cell lung and colorectal cancer, etc. [12, 13, 19]. In line with these previous finding, our data reveal that SUZ12 is significantly elevated in a large subset of human TSCC, thus supporting the notion that SUZ12 might be an bona fide oncogene fostering tumorigenesis in diverse sites throughout the body including tongue. To the best of our knowledge, this might be the first study to uncover the abnormal expression pattern of SUZ12 in TSCC. However, due to the limited number of patients enrolled here, more number of patients from multiple institutions is needed to definitively establish the overexpression pattern of SUZ12 as well as its diagnostic utility in tongue cancer.

Accumulating evidence has demonstrated that SUZ12 overexpression significantly associated with aggressiveness in multiple human cancers [13]. For example, elevated SUZ12 is significantly associated with tumor size, lymph node metastasis and clinical stages in non-small cell lung cancer and colorectal cancer [13, 14]. Similarly, our findings reveal that SUZ12 overexpression significantly associates with cervical lymph nodes metastasis in TSCC, while the associations between other clinicopathological parameters and SUZ12 don’t reach statistical significance. Noticeably, Our findings and others point to an interesting link and positive association between SUZ12 expression and cancer metastasis irrespective of cancer origin and primary site [13, 22, 23]. Complementary to this notion, previous studies have demonstrated that the metastasis-regulator Snail recruits SUZ12 to the CDH1 promoter and represses E-cadherin expression, thus in turn triggers EMT and cancer metastasis [24, 25]. Moreover, targeting SUZ12 by gene knockdown suppresses the migratory and invasive properties of cancer cells and inhibits tumor metastasis in animal models [13, 25]. Taken together, we propose that SUZ12 is a novel cancer biomarker of TSCC which significantly associates with cervical node metastasis. It remains an interesting question to determine the expression level of SUZ12 in the metastatic lesions and its clinical significance, and further unravel its associated mechanistic underpins during cancer metastatic spread.

The past decades have witnessed tremendous progress in diagnosis and treatment for TSCC. However, the 5-year survival rate remains disappointing, suggesting that accurate prognostic prediction is a great challenge but highly beneficial in the clinic [3]. Previous studies have revealed that SUZ12 expression associates with patients’ prognosis and is identified as a key independent prognostic predictor for patients with epithelial ovarian cancer, non-small cell lung cancer and gastric cancer [12, 13]. For example, Li et al. [12] have reported that expression of SUZ12 positively correlates with Ki67 and predicts shorter overall survival in patients with ovarian cancer. Consistent with these findings, our results indicate that patients with high SUZ12 have significantly shorter survival as compared to those with low SUZ12. Furthermore, SUZ12 expression is identified as an independent prognostic factor affecting survival of patients with TSCC. Thus, the expression status of SUZ12 might offer valuable information about patients’ prognosis and indication for effective follow-up management.

A line of evidence has uncovered that SUZ12 is critically involved in tumorigenesis by promoting cell proliferation, migration and metastasis while suppressing apoptosis [12, 13, 25]. Moreover, SUZ12 together with miR-200b is important for cancer stem cell growth and invasive ability in breast cancer cells [26]. In particular, pharmacologic or genetic disruption of SUZ12 inhibited cell proliferation and invasion, and disrupted the maintenance of cancer stem cell both in vitro and in vivo [12, 13, 26, 27]. These abovementioned findings strongly suggest that SUZ12 serves as not only a novel cancer biomarker with diagnostic and prognostic values, but also a viable therapeutic target. Therefore, it remains an open and interesting question to unravel the roles of SUZ12 and its regulatory network during tongue tumorigenesis and to develop efficient approaches to therapeutically targeting SUZ12 in cancer.

## Conclusions

In conclusion, our data reveal that SUZ12 is aberrantly overexpressed in a significant fraction of TSCC and might serve as a novel cancer biomarker as well as therapeutic target for tongue cancer. More studies are warranted to further unravel the mechanistic insights of SUZ12 dysregulation during the initiation and progression of TSCC.

## Abbreviations

SUZ12: suppressor of zest 12
PRC2: polycomb repressive complex 2
TSCC: tongue squamous cell carcinoma
HPV: human papillomavirus
OSCC: oral squamous cell carcinoma
PcG: polycomb group
H3K27me3: trimethylation on lysine 27 residue of histone H3
HR: hazard ratio
95% CI: 95% confidence interval
